# EZH2 promotes angiogenesis in peritoneal dialysis by epigenetically activating SP4 expression in the IL-6/sIL-6R signalling pathway

**DOI:** 10.7150/ijms.78428

**Published:** 2023-01-01

**Authors:** Nan Zhu, Haochen Guan, Xuan Wang, Yueyue Zhang, Lijie Gu, Jieshuang Jia, Ling Wang, Weijie Yuan

**Affiliations:** 1 Department of Nephrology, Shanghai General Hospital, Shanghai, China.; 2 Department of Nephrology, The First Affiliated Hospital of USTC, Division of Life Sciences and Medicine, University of Science and Technology of China, Anhui, China.

**Keywords:** angiogenesis, IL-6/sIL-6R, EZH2, SP4, VEGF, peritoneal dialysis

## Abstract

**Background:** Interleukin-6 (IL-6)/soluble IL-6 receptor (sIL-6R) promotes peritoneal angiogenesis by stimulating SP4-mediated vascular endothelial growth factor (VEGF) production in peritoneal dialysis (PD). Moreover, histone methyltransferase enhancer of zeste homologue 2 (EZH2) is involved in IL-6/sIL-6R signalling via the acceleration of vascular endothelial growth factor (VEGF)-induced angiogenesis. However, the molecular mechanism underlying how EZH2 epigenetically activates VFGF expression in IL-6/sIL-6R signalling during PD is still unclear.

**Methods and Results:** In this study, we measured the expression of EZH2, DNMT3B and SP4 in human peritoneal mesothelial cells (HPMCs) treated with IL-6/sIL-6R stimulation and/or EZH2 overexpression, silencing or inhibition. Methylation of the CpG site in the SP4 promoter region and VEGF production were measured under these treatments in HPMCs. Moreover, tube formation in human umbilical vein endothelial cells (HUVECs) was detected following treatment with conditioned media from these stimulated HPMCs. The 5/6 nephrectomy (5/6Nx) rat model was established, and the rats were injected with peritoneal dialysate. EZH2, DNMT3B and SP4 expression and microvessels were analysed in 5/6Nx + PD rats treated with IL-6/sIL-6R and EZH2 overexpression. The results showed that IL-6/sIL-6R and EZH2 overexpression enhanced the expression of EZH2, DNMT3B and SP4, but EZH2 silencing/inhibition reduced these expression levels. The results for VEGF production and tube formation *in vitro* followed the same trend. IL-6/sIL-6R and EZH2 overexpression increased the methylation percentage of the -170 bp CpG site in the SP4 promoter region in HPMCs. Moreover, IL-6/sIL-6R and EZH2 overexpression stimulated EZH2, DNMT3B and SP4 expression and promoted angiogenesis in 5/6Nx + PD rats.

**Conclusions:** Thus, this study indicated that EZH2 is involved in IL-6/sIL-6R signalling and epigenetically regulates SP4 expression, thereby stimulating VEGF production and angiogenesis in PD. Targeting EZH2 is expected to be a novel therapeutic approach for end-stage renal disease (ESRD) patients with PD treatment.

## Introduction

The incidence of end-stage renal disease (ESRD), which has a high risk of morbidity and mortality, is increasing rapidly worldwide 1. PD is a widely used technique for ESRD treatment and has been proven to be efficient in reducing therapy-related complications, allowing patients to survive longer periods 2. Ultra-filtration failure (UFF), which can have various causes, is the main complication of long-term PD, and it can force ESRD patients to withdraw from PD 2. Accumulating studies have indicated that angiogenesis accompanied by long-term exposure to PD fluid is the main cause of UFF 3.

VEGF is recognized as an important proangiogenic factor on endothelial cells, promoting vascular endothelial cell proliferation and migration in existing vessels and stimulating angiogenesis 4. It has been suggested that the number of peritoneal interstitial vessels in patients with PD is positively correlated with VEGF production 5. Peritoneal angiogenesis was associated with an upregulation of VEGF in mice, while VEGF inhibition reduced PD-induced angiogenesis 6. VEGF can be produced by the local peritoneal tissue of patients on PD. Specific protein (SP)1-SP4 transcription factors play key roles in angiogenesis and VEGF expression 7, 8. Bioinformatics analyses indicated that the VEGF promoter regions contain binding sites for SP1 and SP4. Subsequently, SP4 was found to transcriptionally regulate VEGF expression by binding to its promoter, and silencing of SP4 blocked VEGF-induced angiogenic endothelial tube formation 9.

IL-6 is considered to be a proangiogenic marker that stimulates angiogenesis 10 and thus in patients on PD, it can act as a biomarker of peritonitis related to the peritoneal solute transport rate 11, 12. The combination of IL-6 and sIL-6R induces VEGF expression and secretion to target the binding of nuclear transcription factor specific protein 4 (SP4) to stimulate peritoneal angiogenesis 13. As such, blockade of IL-6R signalling or IL-6R interference arrests angiogenesis *in vitro* and *in vivo* 14. IL-6/sIL-6R combination stimulation promoted angiogenic endothelial tube formation via SP4-mediated VEGF production in HPMCs 9. In addition, recent studies have shown that IL-6/sIL-6R signalling is closely associated with epigenetic regulation in the process of tumorigenesis 15. EZH2 is one of the key epigenetic enzymes whose expression is regulated by IL-6/sIL-6R signalling 16. EZH2 is a histone methyltransferase that acts as an enzymatic catalytic subunit in polycomb repressive complex 2 (PRC2), and it alters gene expression by methylating lysine-27 of histone 3 (H3K27) in nucleosomes 17. Moreover, it interacts with de novo DNA methyltransferases (DNMTs) to methylate the CpG islands of target genes, inducing chromosome structural changes and altering gene transcription 18, 19. EZH2 is closely related to tumour angiogenesis, and inhibition of EZH2 restored normal angiogenesis in endothelial cells 20. EZH2 inhibitors have been proposed to be antiangiogenic and antimetastatic therapeutics 21. Recent research indicated that EZH2 modulates VEGF secretion in diabetic retinas through histone methylation modifications 22 and promotes tumour development by enhancing VEGF expression in clear cell renal cell carcinoma and non-small cell lung carcinoma 23, 24. Although the function of IL-6/sIL-6R and EZH2 in VEGF production and angiogenesis has been well described, whether IL-6/sIL-6R signalling regulation induces EZH2-mediated epigenetic modification of VEGF production and peritoneal angiogenesis during PD is not yet clear.

In this study, we measured the expression of EZH2, DNMT3B and SP4, the methylation level of CpG sites in the SP4 promoter region, and VEGF production in HPMCs treated with IL-6/sIL-6R or with altered EZH2 expression. Moreover, we established 5/6Nx rats, exposed them to PD, and then treated them with IL-6/sIL-6R stimulation and EZH2 overexpression to measure the expression of these genes. Angiogenesis was analysed *in vitro* and *in vivo*. The results confirmed the involvement of EZH2-regulated epigenetic modification of gene expression in IL-6/sIL-6R signalling and indicated that IL-6/sIL-6R-EZH2 promotes VEGF production and peritoneal angiogenesis in PD by epigenetically activating SP4 expression. These data pave the way for the development of a potential therapeutic method targeting EZH2 in clinical practice.

## Materials and Methods

### Cell culture and treatment

HPMCs were isolated from greater omentum specimens from patients undergoing abdominal surgery in Shanghai General Hospital. Cell culture method and its characterization refer to Catar et al. 25. All experiments were conducted with cells no more than the fifth generation to minimize the number of senescent cells. HPMCs were cultivated in Dulbecco's modified Eagle's medium (DMEM) with 10% foetal bovine serum (FBS; Thermo Fisher Scientific, Waltham, MA, USA), 100 U/mL penicillin, and 100 mg/mL streptomycin (Thermo Fisher Scientific). The cultures were incubated at 37 °C in a humidified CO_2_ (5%) atmosphere. The HPMCs were treated with 100 ng/ml IL-6 + sIL-6R for 24 hours and 50 µM GSK126 and 10 µM DZNEP for 6, 12 or 24 hours prior to the subsequent assays. The final volume was 200 µl, and the GSK126 DZNEP control group was treated with DMSO alone.

### Plasmid construction and cell transfection

siRNAs against EZH2 were designed and synthesized by Biostorms (Suzhou, China). The siRNA sequence against EZH2 was GGAUGGUACUUUCAUUGAAGA UUCAAUGAAAGUACCAUC CUG. HPMCs were transiently transfected with EZH2 siRNA by applying the Lipofectamine 2000 transfection reagent (Thermo Fisher Scientific) following the manufacturer's instructions. The cells were then harvested to observe the knockout efficiency via quantitative real-time polymerase chain reaction (qRT-PCR) 48 hours after transfection.

### Tube formation assay

For the tube formation assay, Matrigel (Corning, Tewksbury, MA) was poured onto a 96-well plate (50 ml per well) and solidified at 37 °C for 30 minutes. Human umbilical vein endothelial cells (HUVECs) were purchased from Genomeditech (Shanghai). Co., Ltd (2×10^4^ cells per well) and seeded onto the Matrigel and cultured in MCDB131 medium (Thermo Fisher Scientific, Waltham, USA) with or without 10% (vol/vol) conditioned medium from HPMCs treated as described in the figures. Capillary networks of tubes formed were photographed under a microscope (Zeiss Axiovert 40 CFL, Zeiss, Oberkochen, Germany), and five randomly selected fields from each well were analysed for total capillary length using Image J software (V1.8.0.172, National Institutes of Health).

### Western blotting

The collected cells and rat peritoneal tissues were extracted using protein lysis buffer (Sigma-Aldrich, USA) and quantified via a bicinchoninic acid assay (Pierce, USA). Protein samples (20 µg each sample) were then electrophoresed by 10% sodium dodecyl sulfate-polyacrylamide gel electrophoresis (SDS-PAGE) and transferred to a polyvinylidene difluoride membrane (PVDF, EMD Millipore, MA, USA), which was probed with antibodies against EZH2 (Abcam, ab283270), DNMT3B (Abcam, ab2851) and SP4 (Abcam, ab151777) at a dilution of 1:1000. Blots were subsequently detected and visualized using an enhanced chemiluminescence detection kit (Millipore, Billerica, MA, USA) according to protocols provided by the manufacturer. A Bio-Rad scanning system was used to detect immunoreactive protein bands, and GAPDH was used as a control.

### Stage IV-V chronic kidney disease (CKD) specimens and RNA extraction

Whole blood was collected from Stage IV-V CKD patients and controls with normal kidneys. These specimens were harvested from the Department of Nephrology of Shanghai General Hospital (Shanghai, China) in 2020-2022. All specimens were immediately snap-frozen in liquid nitrogen. This study was conducted with written informed consent obtained from the individual patients and approved by the Ethics Committee of Shanghai Jiaotong University Shanghai Cancer Center. The total RNA in whole blood were extracted according to the protocol of whole blood extraction kit (Sangon Biotech, Shanghai, China).

### qRT-PCR

Total RNA was isolated from cultured HPMCs or rats using TRIzol® reagent (Invitrogen) according to the manufacturer's instructions. The amount of RNA was quantified by measuring the absorbance at 260 nm in triplicate. Quantitative real-time PCR was performed using the iCycler System (Bio-Rad) and Power SYBR Green PCR Master Mix (TaKaRa) as reagents. Relative levels of gene expression were normalized to the Actin gene using the comparative Ct method according to the manufacturer's instructions. The primer sequence information was showed in Supplementary Table 1.

### Enzyme-linked immunosorbent assay (ELISA)

Intracellular VEGF-A release was detected using an ELISA kit (Solarbio, Beijing) according to the manufacturer's instructions.

### Immunostaining

Cultured HPMCs were fixed in 4% paraformaldehyde and permeabilized with 0.25% Triton X-100. After blocking in 2% BSA (Invitrogen) for 30 min, the cells were incubated with primary antibodies at 4 °C overnight and then incubated with FITC-conjugated anti-rabbit secondary antibody (1:1000, Invitrogen) and Cy3-conjugated anti-mouse secondary antibody (1:1000, Jackson ImmunoResearch). Nuclei were stained with 4,6-diamidino-2-phenylindole (DAPI, 1:5000, Sigma) for 5 min. Positive staining was observed under a fluorescence microscope (Olympus, Japan).

### Animal model of 5/6 subtotal nephrectomy

All animal procedures were performed under appropriate licences and according to institutional animal care guidelines and approved by the institutional ethics committee of Shanghai General Hospital. The rats were purchased from Vital River Laboratory Animal Technology (Beijing, China). All animals were housed in individual ventilated cages, provided sterilized water and food ad libitum and handled under specific pathogen-free conditions in the institute's animal care facilities, which meet international standards. Rats underwent 5/6Nx or sham surgery under ketamine/xylazine anaesthesia (100 mg/kg) and sterile conditions. Briefly, the left kidney was exposed, and the upper and lower poles were tied with a polyglycolic acid suture line, followed by right nephrectomy. The peritoneum and skin were then sutured, and the animals were returned to their individual cages.

### Preparation of EZH2 expression vectors and injection

A vector for the overexpression of EZH2 was obtained from Biostorms (Suzhou, China). Chemical modifications of pre-EZH2 were included for use in selection and to improve the stability of the guide chain. A negative control was prepared using a nonsense oligonucleotide. The lentivirus (10^9^ TU/mL) or its control (10^9^ TU/mL) was mixed with cationic lipid polystyrene (4 μg/μL) and cultured for 15 minutes at 37 °C. The sham and 5/6Nx rats were injected with 20 ml of peritoneal dialysate (3.86% glucose) daily for 4 weeks. Then, a 7-μL aliquot of the mixture and IL-6/sIL-6R (100 mg/kg/day) were injected into the 5/6Nx + PD rats. The control group was intraperitoneally injected with normal saline.

### Immunohistochemistry (IHC)

Paraffin-embedded blocks were cut into 4-μm-thick sections and dewaxed and hydrated. Then, the sections were immersed in distilled water containing 3% hydrogen peroxidase twice to reduce endogenous oxidase activity. The peritoneal sections were incubated with anti-CD34 antibody (Abcam, ab185732) at a dilution of 1:1000 at 4 °C for 18 hours, washed with 0.1 M PBS and incubated with goat anti-rabbit antibodies (Abcam, ab6721) at a 1:5000 dilution at room temperature for 1 hour. The sections were then washed with 0.1 M PBS for 5 minutes 3 times. After the chromogenic reaction, the sections were placed under a cover slip and observed under a microscope.

### DNA methylation analysis

To conduct the differential analysis of DNA methylation at the CpG sites in SP4 promoter regions, the levels of DNA methylation and RNA were quantified in cell samples. Genomic DNA was extracted from HPMCs using a QIAamp DNA mini-kit (Qiagen) and digested with HindIII overnight. The DNA was denatured (99 °C, 5 min), incubated in 0.3 M NaOH (39 °C, 5 min) and treated with bisulfite (4 M sodium bisulfite, 6 μM hydroquinone, 0.3 M guanidine HCl) at 55 °C for 16 hr. Bisulfite-treated genomic DNA was purified using the Wizard kit (Promega) and desulfonated in 0.3 M NaOH (37 °C, 15 min); desulfonation was terminated by the addition of NH4OAc, and DNA was coprecipitated with 20 μg of linear acrylamide in EtOH overnight at -20 °C. Amplification of the SP4 promoter was performed by nested PCR according to the manufacturer's protocol. PCR products were cloned into the pGEM-T-easy plasmid (Promega), and 12-18 minipreps from 2-3 independent PCRs/ligations were randomly selected and sequenced to quantify DNA methylation in each sample.

### Statistical analyses

Statistical analysis was performed using GraphPad Prism 6.05 software (GraphPad Software). The data were analysed with the t test or repeated-measures ANOVA for paired data (*in vitro* experiments) or unpaired data (animal experiments) as appropriate. The results are expressed as the means ± SEM. Findings with a P value of 0.05 were considered significant.

## Results

### EZH2 is involved in IL-6/sIL-6R signalling and regulates the expression of DNMT3B and SP4 in HMPCs

**Figure 1:**
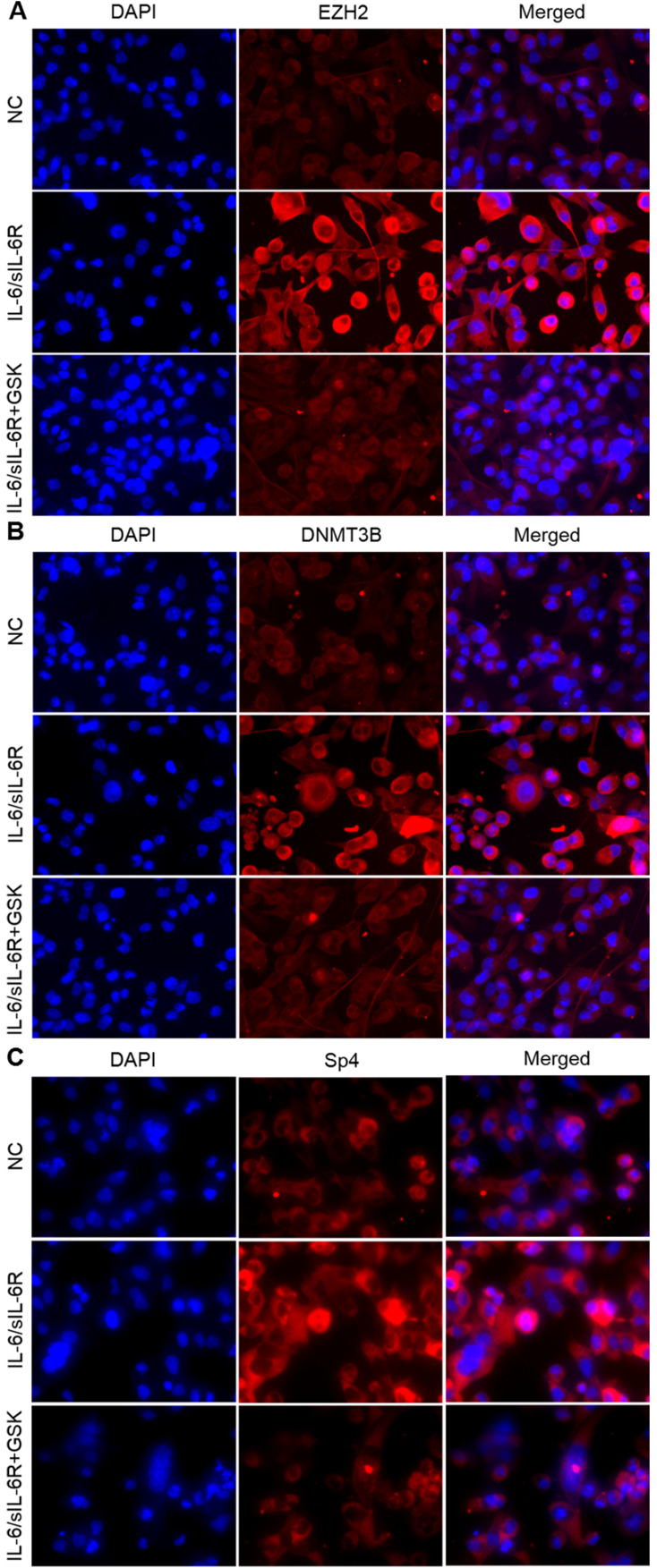
EZH2 is involved in IL-6/sIL-6R signalling and regulates DNMT3B and SP4 expression in HMPCs. Immunofluorescence staining results show EZH2 **(A)**, DNMT3B** (B) a**nd SP4** (C)** expression in HMPCs under IL-6/sIL-6R and IL-6/sIL-6R + GSK stimulation.

To identify the role of IL-6/sIL-6R stimulation in EZH2, DNMT3B and SP4 expression, we carried out immunofluorescence analysis in HMPCs. The results showed that the expression levels of EZH2, DNMT3B and SP4 were obviously increased with IL-6/sIL-6R treatment compared to the normal controls (Fig. 1A-C). These expression levels were visibly reduced when treated with GSK 126 (an EZH2 inhibitor, referred to as GSK in the remainder of the text) and IL-6/sIL-6R (Fig. 1A-C). These results suggested that EZH2 is involved in IL-6/sIL-6R signalling and regulates DNMT3B and SP4 expression in HMPCs.

**Figure 2:**
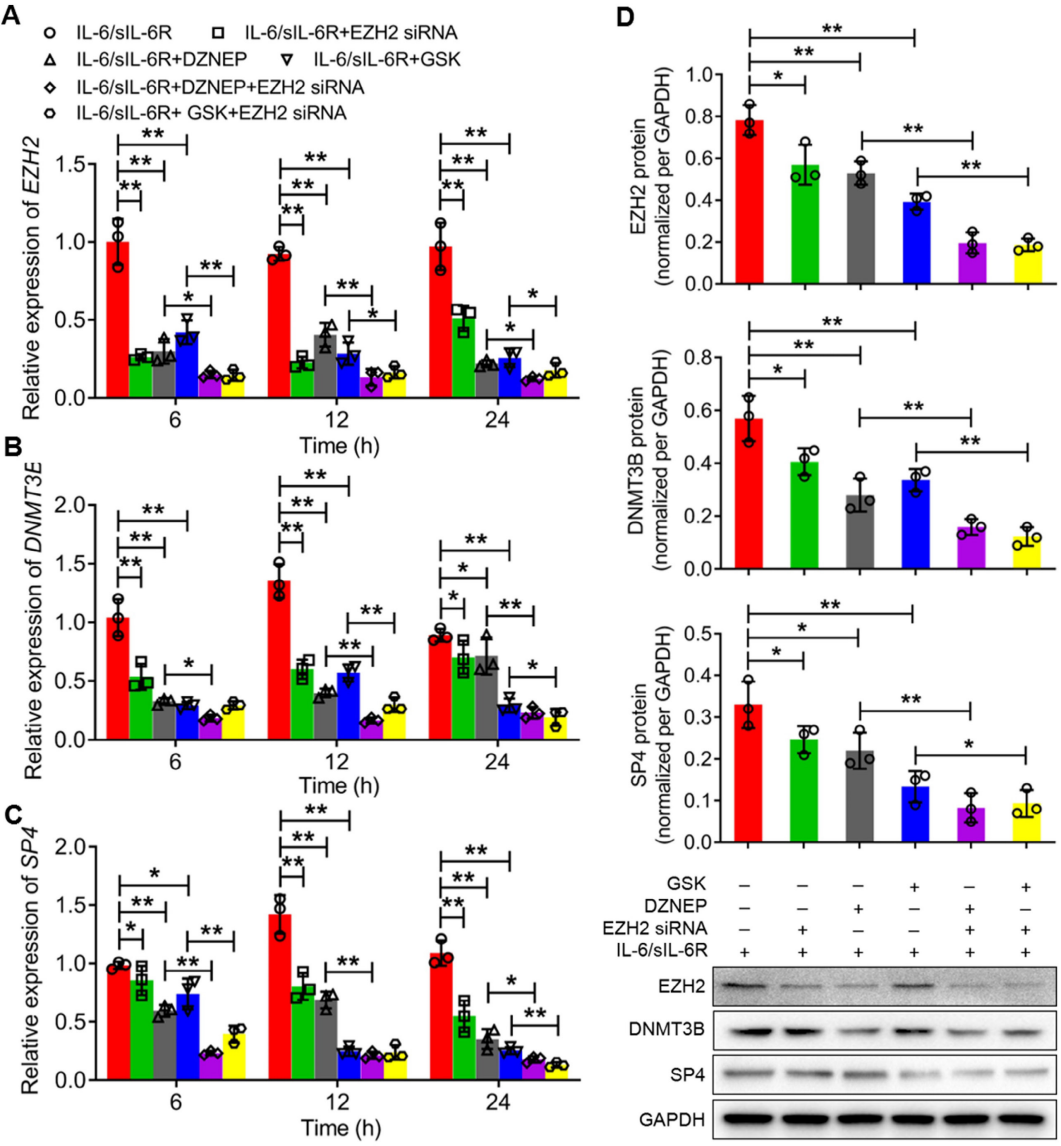
** IL-6/sIL-6R-EZH2 signalling regulates the expression of DNMT3B and SP4 in HMPCs.** The mRNA expression levels of EZH2** (A)**, DNMT3B **(B)** and SP4 **(C)** in HMPCs under IL-6/sIL-6R and EZH2 overexpression treatment for 6 h, 12 h or 24 h. **(D)** The protein expression levels of EZH2, DNMT3B and SP4 in HMPCs after 24 h of IL-6/sIL-6R, EZH2-OE and IL-6/sIL-6R + EZH2-OE treatment and the quantitative analysis of the western blotting results. The final concentration of IL-6/sIL-6R treatment was 100 ng/ml. NC, normal control. The data are represented as the means + SDs, n ≥ 3; * P < 0.05, ** *P* < 0.01.

**Figure 3:**
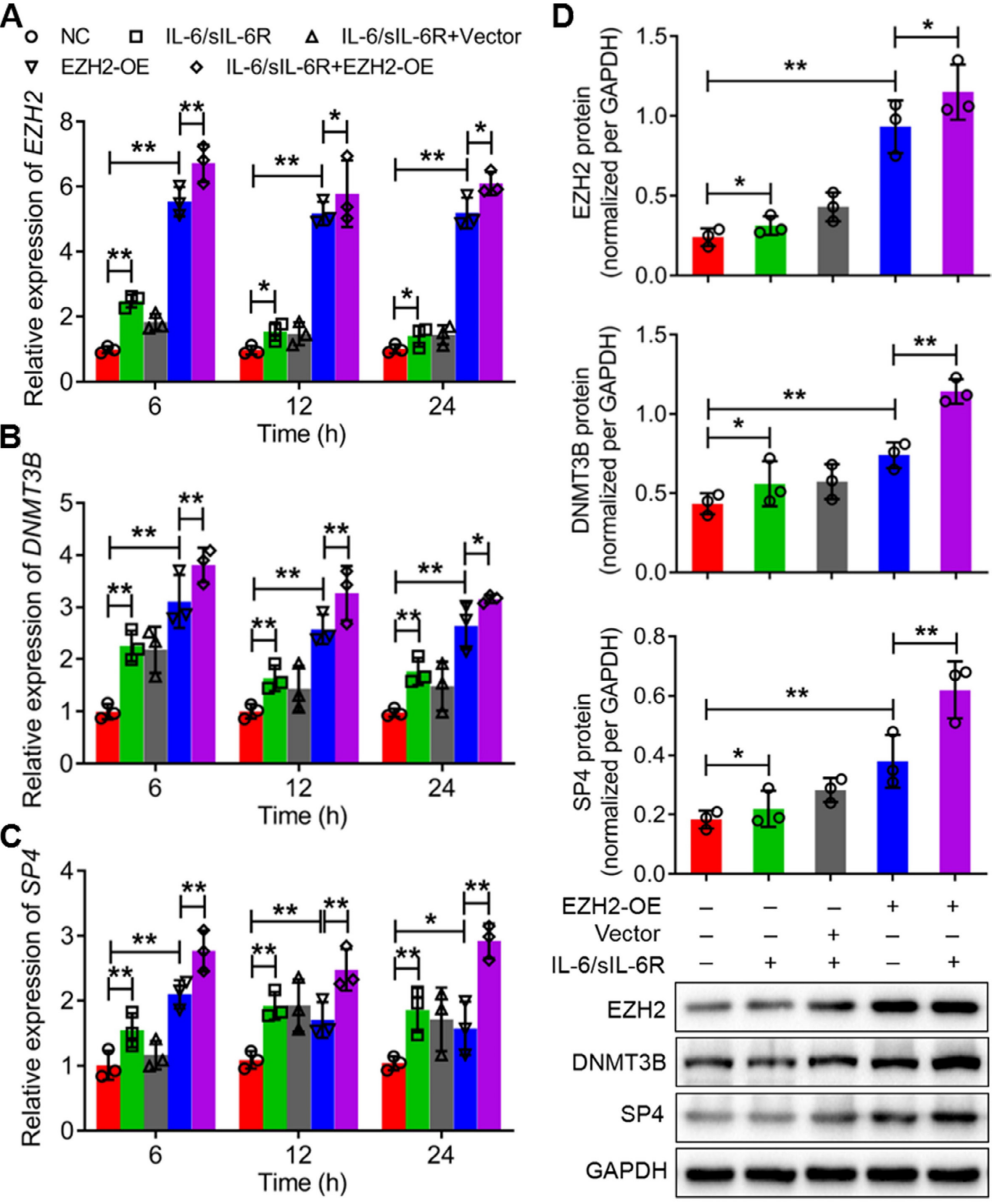
** IL-6/sIL-6R-EZH2 signalling regulates the expression of DNMT3B and SP4 in HMPCs.** The mRNA expression levels of EZH2 **(A)**, DNMT3B **(B)** and SP4 **(C)** in HMPCs with IL-6/sIL-6R, IL-6/sIL-6R + EZH2 siRNA, IL-6/sIL-6R + DZNEP, IL-6/sIL-6R + GSK, IL-6/sIL-6R + DZNEP + EZH2 siRNA and IL-6/sIL-6R + GSK + EZH2 siRNA treatments for 6 h, 12 h or 24 h. **(D)** Western blot analysis showed the protein expression levels of EZH2, DNMT3B and SP4 in HMPCs with the corresponding treatments for 24 h. The final concentration of the IL-6/sIL-6R treatment was 100 ng/ml. The data are represented as the means + SDs, n ≥ 3; * P < 0.05, ** *P* < 0.01.

The expression levels of EZH2, DNMT3B and SP4 were further analysed through qRT-PCR and western blotting after treatment with IL-6/sIL-6R in EZH2-overexpressing or EZH2-silenced HPMCs. The qRT-PCR results showed that the mRNA levels of EZH2, DNMT3B and SP4 increased significantly with 6 h of IL-6/sIL-6R stimulation compared to the normal controls, which was consistent with the results after 12 h or 24 h of IL-6/sIL-6R treatment (Fig. 2A-C). Meanwhile, EZH2-OE increased these expression levels significantly compared to the normal controls. Moreover, expression levels increased even more when treated with IL-6/sIL-6R + EZH2-OE compared only EZH2-OE treatment for 6 h, 12 h or 24 h (Fig. 2A-C). The western blotting results after 24 h of the different treatments were consistent with the qRT-PCR results (Fig. 2D). Conversely, EZH2 siRNA, DZNEP (a PRC2 inhibitor) and GSK significantly reduced the expression of EZH2, DNMT3B and SP4 after 6 h, 12 h or 24 h of IL-6/sIL-6R treatment. Expression levels were decreased to even lower levels with IL-6/sIL-6R + DZNEP + EZH2 siRNA and IL-6/sIL-6R + GSK + EZH2 siRNA treatment (Fig. 3A-C). The western blotting results confirmed the qRT‒PCR results completely (Fig. 3D). Taken together, these results indicated that EZH2 is involved in IL-6/sIL-6R signalling and that EZH2 plays key roles in the regulation of DNMT3B and SP4 expression in HMPCs.

### IL-6/sIL-6R-EZH2 signalling increased the methylation level of the SP4 promoter regions in HMPCs

**Figure 4:**
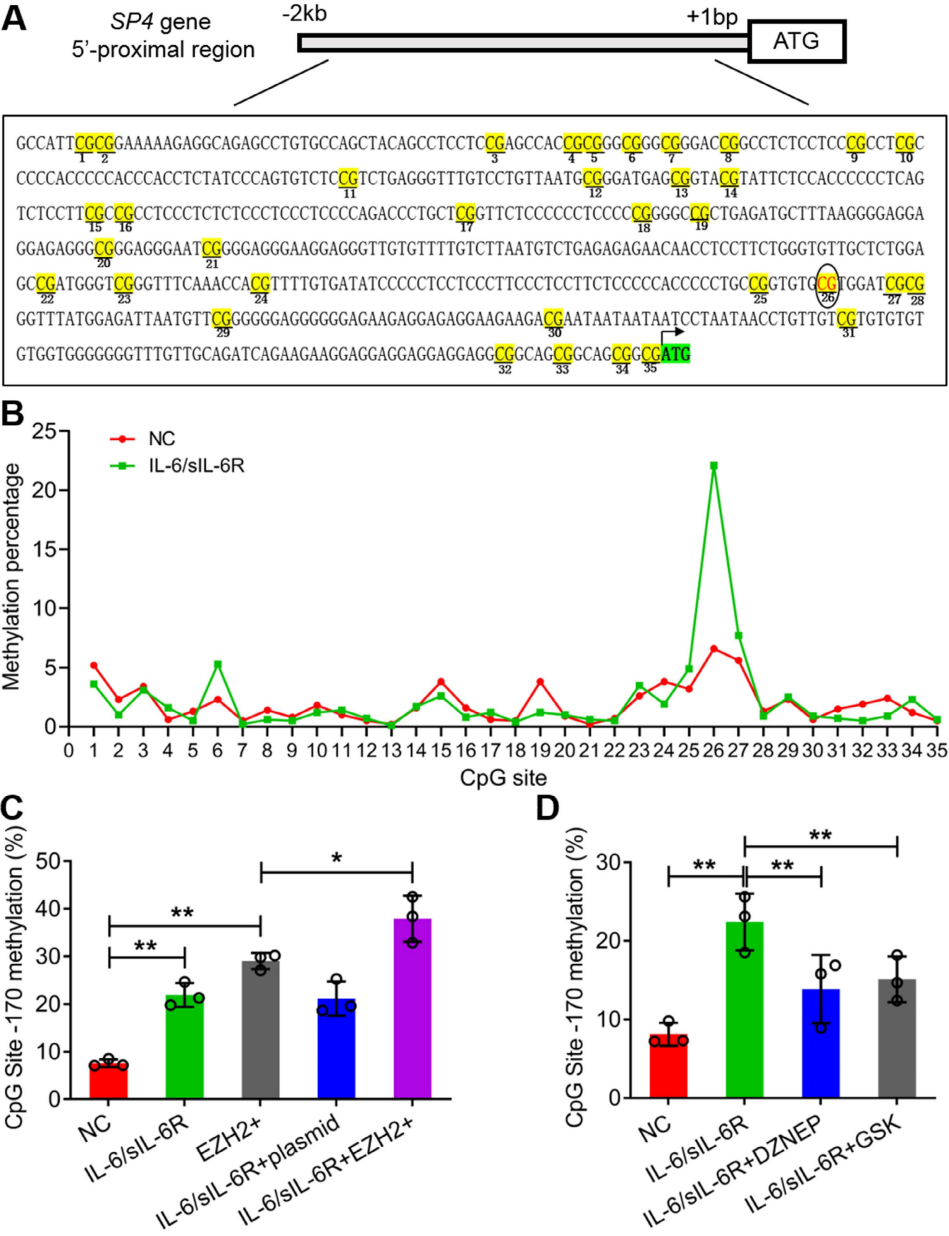
** IL-6/sIL-6R-EZH2 signalling modifies the DNA methylation of the CpG site (-170 bp) in the SP4 promoter region in HMPCs. (A)** The 35 CpG sites in the SP4 promoter region (2 kb upstream of the ATG site). **(B)** The methylation percentage of the 35 CpG sites stimulated by IL-6/sIL-6R in HMPCs. **(C, D)** The methylation percentage of the 26th CpG site (-170 bp) in the SP4 promoter region in IL-6/sIL-6R, EZH2 overexpression and EZH2 inhibition conditions in HMPCs. The data are represented as the means + SDs, n ≥ 3; * P < 0.05, ** *P* < 0.01.

According to sequence analysis, 35 CpG sites were detected in the 2 kb sequence upstream of the ATG of SP4 (Fig. 4A). IL-6/sIL-6R treatment obviously increased the methylation percentage of the 26th CpG site (-170 bp); the value was more than 20% in the IL-6/sIL-6R group but less than 10% in the control (Fig. 4B). To detect whether EZH2 functions in the methylation of this CpG site, we analysed the methylation of this site with EZH2 overexpression and inhibition. The results showed that EZH2 overexpression and IL-6/sIL-6R treatment alone significantly increased the methylation percentage of this site, and IL-6/sIL-6R + EZH2 overexpression combination treatment increased this level even more strongly (Fig. 4C). Conversely, DZNEP and GSK reduced the methylation level of this site after IL-6/sIL-6R treatment (Fig. 4D). These results indicated that IL-6/sIL-6R-EZH2 modifies the CpG methylation of the SP4 promoter region in HMPCs.

### IL-6/sIL-6R-EZH2 regulates VEGF production and angiogenesis in HMPCs

**Figure 5:**
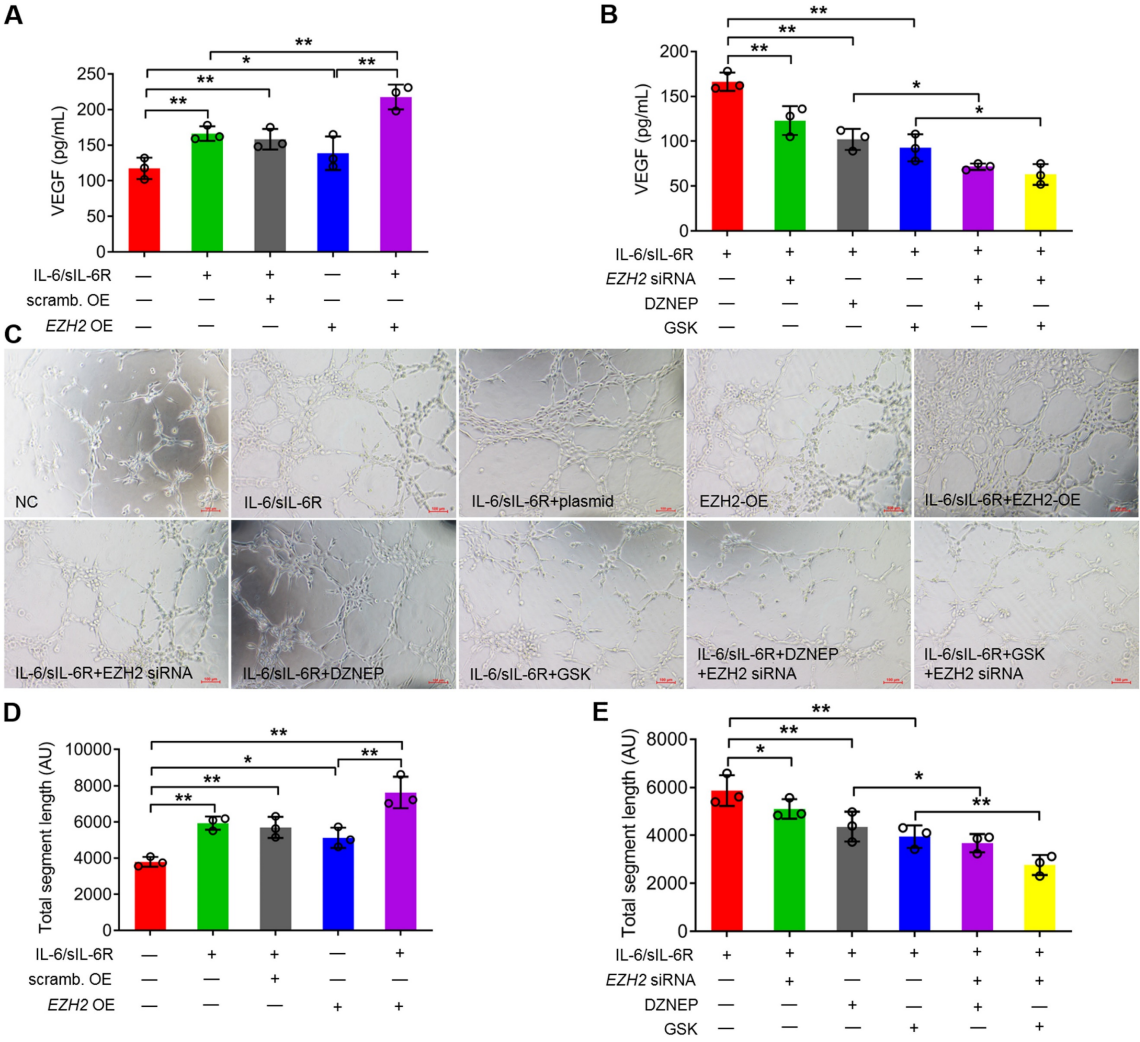
** IL-6/sIL-6R-EZH2 signalling promotes angiogenesis in HUVECs. (A)** VEGF secretion was assessed in HPMCs treated with IL-6/sIL-6R and EZH2-OE compared to the NC or scrambled control OE groups for 24 h. **(B)** VEGF secretion was assessed in HPMCs treated with IL-6/sIL-6R and EZH2 siRNA, DZNEP, GSK, EZH2 siRNA + DZNEP, or EZH2 siRNA + GSK for 24 h. **(C)** Endothelial cell tube formation in HUVECs treated with conditioned medium from A and B for 16 h. **(D, E)** Quantification of the total segment lengths in C. The data are represented as the means + SDs, n ≥ 3; * P < 0.05, ** *P* < 0.01.

To explore the role of EZH2 in angiogenesis, VEGF production and tubule formation assays were analysed. In HMPCs, VEGF production was significantly increased under IL-6/sIL-6R treatment and was also significantly increased in the EZH2-overexpressing group compared to the normal controls. The VEGF level was significantly higher in the IL-6/sIL-6R + EZH2-OE treatment group than in the IL-6/sIL-6R or EZH2-OE group (Fig. 5A). In contrast, EZH2 siRNA + IL-6/sIL-6R treatment resulted in a significant reduction in VEGF release compared to that in the IL-6/sIL-6R group (Fig. 5B). Moreover, DZNEP or GSK supplementation in IL-6/sIL-6R treatment revealed similar effects on VEGF release. EZH2 siRNA further reduced the VEGF levels in the IL-6/sIL-6R + DZNEP and IL-6/sIL-6R + GSK groups (Fig. 5B).

To detect the impact of VEGF production, conditioned medium from stimulated HPMCs was transferred to HUVEC cultures, and the formation of capillaries was assessed. Both IL-6/sIL-6R treatment and EZH2 overexpression significantly increased the total segment length in HPMCs compared with the control group. Tubule formation was further stimulated by IL-6/sIL-6R + EZH2-OE combination treatment (Fig. 5C, D). However, the tubule formation of HUVECs was significantly reduced with EZH2 siRNA, DZNEP or GSK treatments with IL-6/sIL-6R stimulation, and EZH2 siRNA reduced tubule formation to even lower levels in the IL-6/sIL-6R + DZNEP and IL-6/sIL-6R + GSK groups (Fig. 5C, E). These results demonstrated that IL-6/sIL-6R-EZH2 signalling regulates VEGF production in HPMCs and exerts a key role in angiogenesis in HUVECs.

### IL-6/sIL-6R-EZH2 promoted peritoneal angiogenesis in 5/6 nephrectomy rats exposed to PD

To clarify the role of EZH2 in peritoneal angiogenesis *in vivo*, a 5/6 nephrectomy rat model was established. Peritoneal hyperplasia increased significantly in the 5/6 nephrectomy rats compared with the sham group, as demonstrated by haematoxylin-eosin (HE) staining (Supplemental Fig. 1).

**Figure 6:**
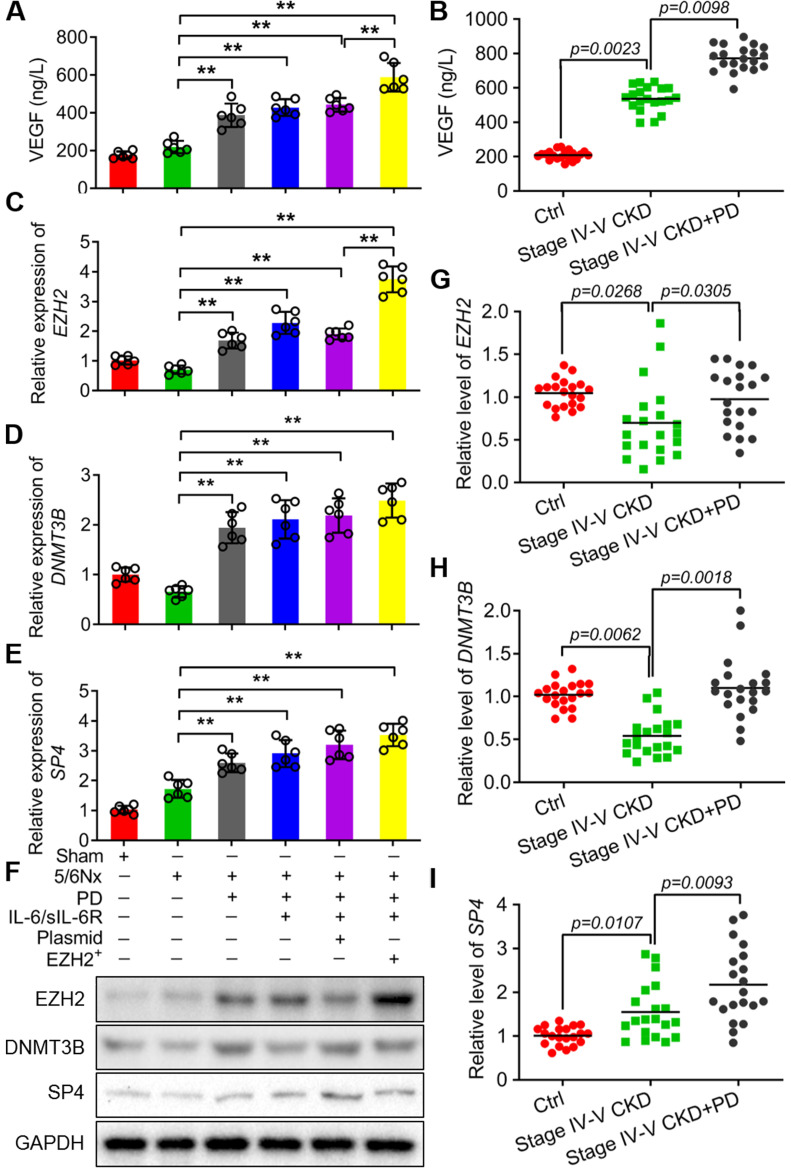
** IL-6/sIL-6R-EZH2 signalling enhanced VEGF secretion and the expression levels of EZH2, DNMT3B and SP4 in 5/6 nephrectomy rats and CKD patients.** VEGF secretion was assessed in 5/6 nephrectomy rats **(A)** and CKD patients** (B)**. **(C-E)** The mRNA expression levels of EZH2 (A), DNMT3B (B) and SP4 (C) in sham, 5/6Nx, 5/6Nx + PD, 5/6Nx + PD + IL-6/sIL-6R and 5/6Nx + PD + IL-6/sIL-6R + EZH2 rats. **(F)** Western blotting analysis showed the protein levels of EZH2, DNMT3B and SP4 in 5/6Nx rats with corresponding treatments in A-C. **(G-I)** The mRNA expression levels of EZH2, DNMT3B and SP4 in stage IV-V CKD patients with PD therapy. The data are represented as the means + SDs, n ≥ 3; * P < 0.05, ** *P* < 0.01.

To further confirm VEGF production was involved in IL-6/sIL-6R-EZH2 signaling pathway. The results showed that the level of VEGF was significantly increased with PD, IL-6/sIL-6R + PD or IL-6/sIL-6R + PD + EZH2 overexpression treatment (Fig. 6A) and CKD patients (Fig. 6B). In addition, the mRNA and protein expression levels of EZH2, DNMT3B and SP4 were all significantly increased with PD, IL-6/sIL-6R + PD or IL-6/sIL-6R + PD + EZH2 overexpression treatment compared to the 5/6Nx treatment (Fig. 6C-F). The results of qRT-PCR from stage IV-V CKD patients showed that EZH2 and DNMT3B mRNA expression was significantly decreased, but SP4 expression was significantly increased compared to the controls. PD therapy significantly increased the expression of these genes compared to that in the CKD groups (Fig. 6G-I).

**Figure 7:**
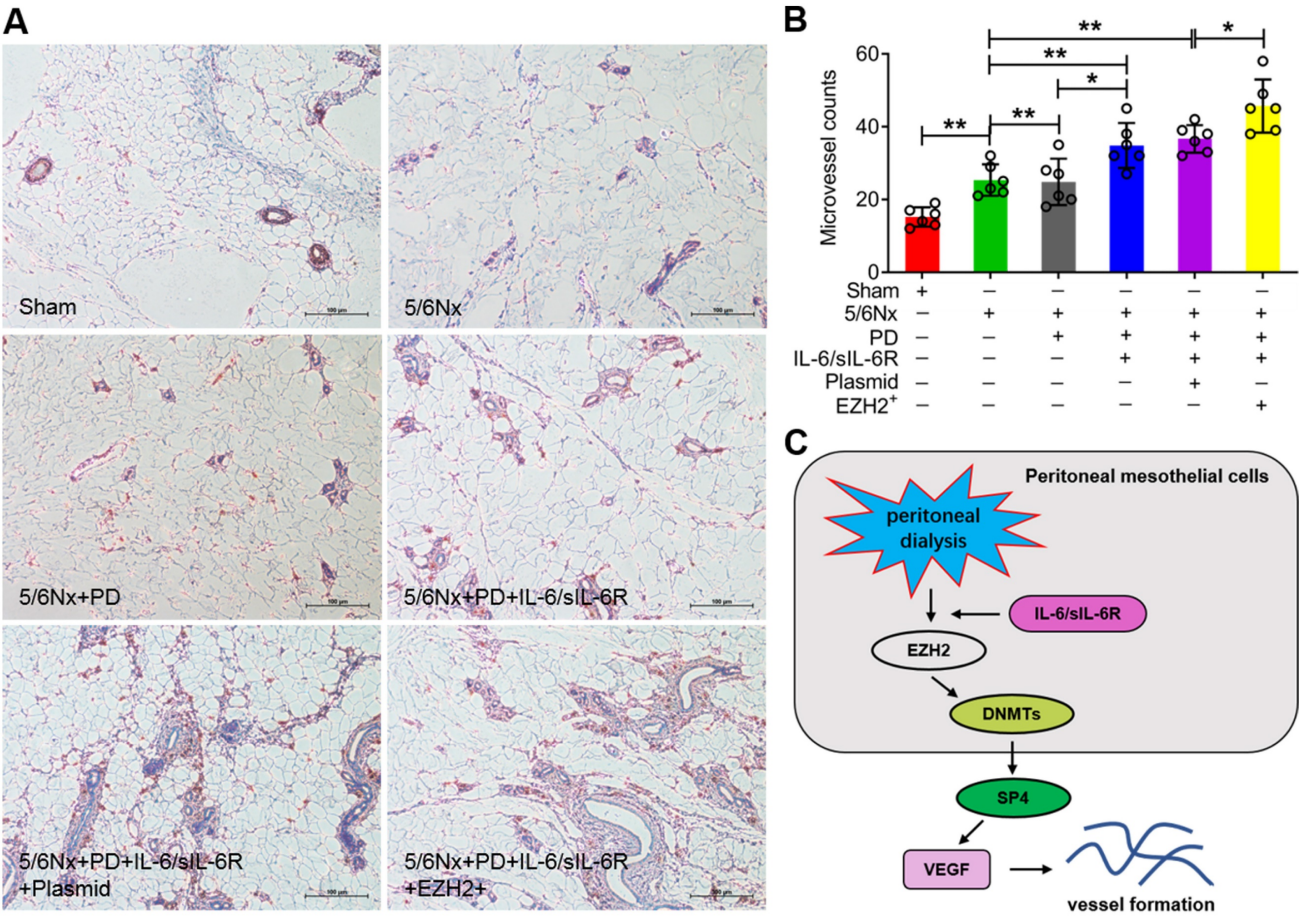
** IL-6/sIL-6R-EZH2 signalling promoted peritoneal angiogenesis in 5/6Nx rats. (A)** Microvessel density was determined by IHC assays in the peritoneum of 5/6Nx rats with PD, IL-6/sIL-6R, IL-6/sIL-6R + PD, and IL-6/sIL-6R + PD + EZH2 treatments and compared to the control sham group. **(B)** Quantitative analysis of the microvessel counts in A.** (C)** Proposed scheme for the mechanism by which IL-6/sIL-6R-EZH2 signalling promotes vessel formation *in vivo* and *in vitro*. Bars = 100 μm. The data are represented as the means + SDs, n ≥ 3; * P < 0.05, ** *P* < 0.01.

Immunohistochemistry was performed to assess positive CD34 signalling. The microvessel counts were significantly increased in the 5/6Nx + PD rats compared to the 5/6Nx group, IL-6/sIL-6R treatment in 5/6Nx + PD rats increased the counts further, and EZH2-OE in the 5/6Nx + PD + IL-6/sIL-6R group increased the counts even higher (Fig. 7A, B).

## Discussion

PD is the most widely used therapy for chronic kidney disease (CKD) and ESRD populations 26. Chronic inflammation and angiogenesis are the most common complications in patients with long-term PD and are associated with peritoneum remodelling and resulting in UFF 27, 28. IL-6 is a main proinflammatory cytokine, and IL-6/sIL-6R stimulation elevated VEGF expression and release through a transcriptional mechanism involving STAT3 and SP4 in HPMCs and promoted angiogenesis in the mouse peritoneal membrane 9. In this study, VEGF production in HPMCs was upregulated, and angiogenic endothelial tube formation in HUVECs was induced by IL-6/sIL-6R treatment, which was consistent with previous findings. Moreover, IL-6/sIL-6R stimulation in 5/6Nx + PD rats significantly promoted microvessel formation. These results indicated that IL-6/sIL-6R plays key roles in VEGF-mediated peritoneal angiogenesis.

The crosstalk between IL-6/sIL-6R signalling and epigenetic regulation is gradually attracting attention. Recently, IL-6/sIL-6R was demonstrated to induce human vascular smooth muscle cells (VSMCs) to differentiate into osteoblast-like cells through JMJD2B-mediated histone demethylation in the RUNX2 promoter 29. It was suggested that IL-6 induced VEGFR2 expression via a promoter methylation-dependent mechanism and caused disordered angiogenesis in endothelial cells 30. In this study, the expression of EZH2 and DNMT3B increased in IL-6/sIL-6R-treated HPMCs, and tube formation was upregulated with IL-6/sIL-6R treatment or EZH2 overexpression. The 5/6Nx +PD + IL-6/sIL-6R rats also showed increased expression, and microvessel formation was stimulated with IL-6/sIL-6R treatment or EZH2 overexpression. Considering EZH2 and DNMT3B are key methyltransferases involved in histone and DNA stability, our data suggests that IL-6/sIL-6R signalling induces epigenetic modifications to promote VEGF-induced angiogenesis, which confirmed previous results.

EZH2 is an H3K27 methyltransferase associated with tumour angiogenesis that has become a significant hallmark of multiple cancers 31. DNMT3B is a DNA methyltransferase that plays important roles in organ development and disease 32, 33. The expression levels of EZH2 and DNMT3B were decreased in stage IV-V CKD patients; however, expression increased under PD treatment, and PD therapy in 5/6Nx rats induced EZH2 and DNMT3B expression significantly, which indicated that epigenetic mechanisms involving EZH2 and DNMT3B might play crucial roles in peritoneum angiogenesis and remodelling in PD. In addition, the expression of DNMT3B and SP4 was upregulated with EZH2 overexpression but reduced by EZH2 silencing or inhibition in HPMCs, which suggested that EZH2 regulates DNMT3B and SP4 activation in the peritoneum. The DNA methylation level of the -170 bp CpG site in the SP4 promoter region was elevated in EZH2-overexpressing or IL-6/sIL-6R-stimulated HPMCs, and the combination treatment increased the value further, which indicated that IL-6/sIL-6R-EZH2 signalling might promote SP4 expression by modifying methylation of its promoter. CpG methylation by DNMT3B is essential for mammalian development and is frequently dysregulated in diseases 34. Considering the role of EZH2 in DNMT3B and SP4 expression and the function of DNMT3B in CpG methylation, EZH2 may trigger SP4 activation by regulating DNMT3B-mediated epigenetic regulation in IL-6/sIL-6R signalling, thereby promoting VEGF production and angiogenesis. However, the precise epigenetic mechanism of how DNMT3B activates SP4 requires further investigation.

In conclusion, this study indicated that IL-6/sIL-6R recruits EZH2/DNMT3B to epigenetically activate SP4 expression, leading to VEGF production and peritoneal angiogenesis (Fig. 7C). Because SP4 expression was significantly increased in 5/6Nx + PD rats, further increased with IL-6/sIL-6R stimulation, and most strongly enhanced by the combination of IL-6/sIL-6R + EZH2 overexpression, we propose that EZH2 might be a key mediator in IL-6/sIL-6R signalling promoting VEGF-induced angiogenesis during PD. Inhibitors targeting EZH2 are expected to be promising drugs for ESRD patients maintaining long-term PD.

## Supplementary Material

Supplementary figure and table.

## Abbreviations

SP4: specific protein 4
VEGF: vascular endothelial growth factor
PD: peritoneal dialysis
ESRD: end-stage renal disease
UFF: Ultra-filtration failure
HPMCs: human peritoneal mesothelial cells
IL-6: interleukin-6
PSTR: peritoneal solute transport rate
sIL-6R: soluble interleukin-6 receptor
HUVECs: human umbilical vein endothelial cells
H3K27: lysine-27 of histone H3
DNMT: DNA methyltransferase
DNMT3B: DNA methyltransferase 3B
