# Supplementary material for: EZH2 promotes angiogenesis in peritoneal dialysis by epigenetically activating SP4 expression in the IL-6/sIL-6R signalling pathway
Source: Int J Med Sci. 2023 Jan 1;20(1):114–24. doi: 10.7150/ijms.78428 (PMC9812808; doi:10.7150/ijms.78428)
Supplement: Supplementary file 1 — Supplementary figure and table. [file ijmsv20p0114s1.pdf]

Table 1 The qRT-PCR primer sequence information.

Gene	Gene ID	Upstream primer（5'-3'）	Downstream primer（5'-3'）
Actin	2934	CATCACTGCCACCCAGAAGACTG	ATGCCAGTGAGCTTCCCGTTCAG
EZH2	2146	GGACTCAGAAGGCAGTGGAG	CTTGAGCTGTCTCAGTCGCA
DNMT3B	1789	CCGCTTCCTCGCAGCAG	CGATCGCCGAGCTAGGTTTA
SP4	6671	AGGTTTATGCGGAGTGATCATCTC	CTCTGTAACAGATGAGTCCAGTT

Supplemental figure 1

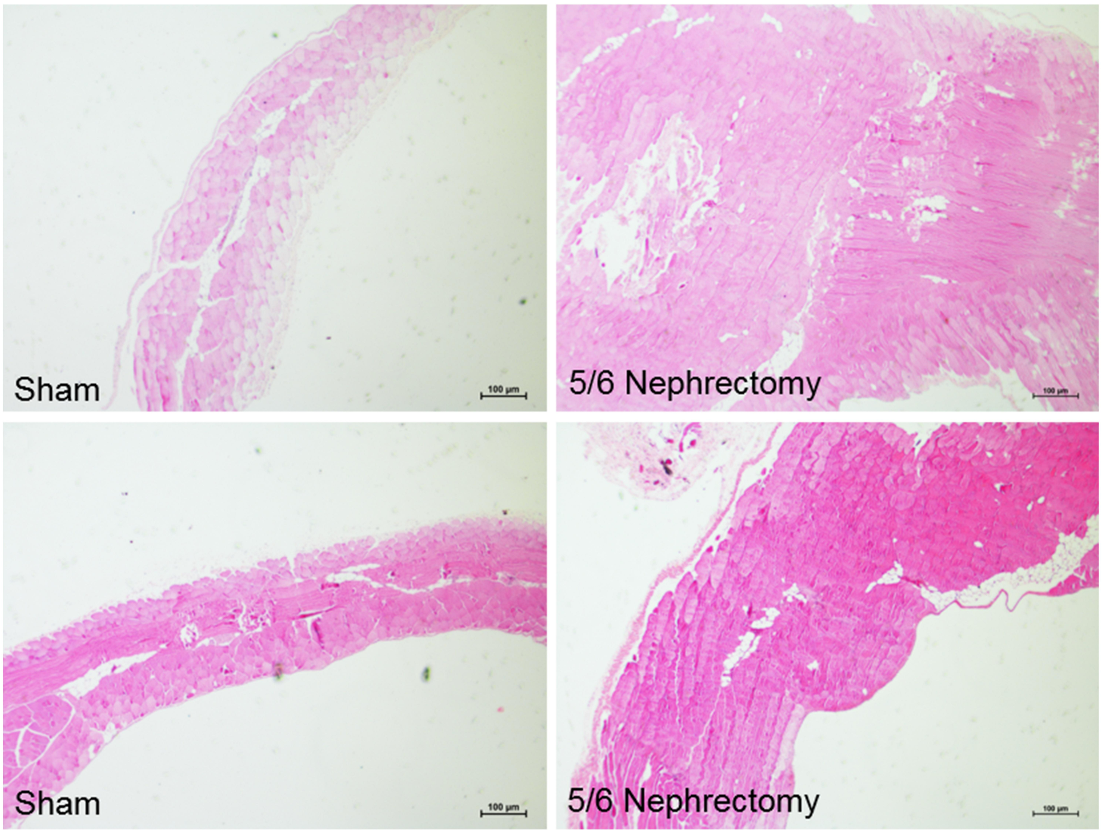

Figure. HE staining. Compared with the sham group, peritoneal hyperplasia increased significantly in the 5/6 nephrectomy rats.
